# The hematopoietic stem-cell niche in health and leukemia

**DOI:** 10.1007/s00018-016-2306-y

**Published:** 2016-07-19

**Authors:** Abel Sánchez-Aguilera, Simón Méndez-Ferrer

**Affiliations:** 1 Stem Cell Niche Pathophysiology Group, Centro Nacional de Investigaciones Cardiovasculares (CNIC), Melchor Fernández Almagro 3, 28029 Madrid, Spain; 2 Wellcome Trust-Medical Research Council Cambridge Stem Cell Institute and Department of Haematology, University of Cambridge, and National Health Service Blood and Transplant, Cambridge Biomedical Campus, Cambridge, CB2 0PT UK

**Keywords:** Hematopoietic stem cell, Stem cell niche, Bone marrow microenvironment, Leukemia, Leukemia stem cell

## Abstract

Research in the last decade has shown that hematopoietic stem cells (HSCs) interact with and are modulated by a complex multicellular microenvironment in the bone marrow, which includes both the HSC progeny and multiple non-hematopoietic cell types. Intense work is gradually throwing light on the composition of the HSC niche and the molecular cues exchanged between its components, which has implications for HSC production, maintenance and expansion. In addition, it has become apparent that bidirectional interactions between leukemic cells and their niche play a previously unrecognized role in the initiation and development of hematological malignancies. Consequently, targeting of the malignant niche holds considerable promise for more specific antileukemic therapies. Here we summarize the latest insights into HSC niche biology and recent work showing multiple connections between hematological malignancy and alterations in the bone marrow microenvironment.

## Introduction

The concept of stem cell niche, in the context of the hematopoietic system, implies that the behavior of hematopoietic stem cells (HSCs) is to a great extent modulated by their microenvironment in the bone marrow. Underlying the interest in the study of the HSC niche are two practical hypotheses: (1) precise characterization of the cell populations and factors produced by them, responsible for HSC maintenance in vivo, holds the clue for HSC expansion ex vivo; (2) knowledge of how the niche is modified in hematological diseases (such as dysplasias and leukemias) might allow selective therapeutic targeting that eliminates abnormal HSCs while restoring normal hematopoiesis. However, the complex cellular composition of the BM cavity, the multiple anatomical and functional interactions between its components—not yet fully characterized—and its apparent lack of physical compartmentalization into discrete, organized structures analogous to stem cell niches described in other tissues have complicated the cellular and molecular dissection of the HSC niche. As a consequence, significant progress in the two above mentioned directions has only begun to occur in very recent years. In this review, we discuss the most recent insights into the biology of HSCs and their niche, some of the technical difficulties that have been encountered, and recent work revealing the relationship between hematological disease and abnormalities of the HSC niche.

## HSCs: current dogmas and evolving concepts

Our idea of the HSC has traditionally been dominated by a number of concepts:

1. A static, long-term HSC with fixed specific features (including a characteristic immunophenotype, quiescent state, self-renewing properties), ultimately responsible for maintaining hematopoiesis through life.
2. A convenient, straightforward, but simplistic model of progenitor hierarchy, in which a homogeneous population of HSC differentiates into progenitors and subsequently mature lineages along bifurcating, non-reversible, non-overlapping paths.
3. A rigid idea of a “HSC niche”, in which—by analogy with certain non-hematopoietic tissues—the HSC is postulated to “reside” more or less statically in discrete anatomical microenvironments, the exact identity of which has remained elusive. The growing amount and complexity of information concerning the influence of many other bone marrow cells on HSC behavior has lead to postulate the existence of functionally different HSC niches, which is still not sufficiently substantiated by the experimental data available [1].

In the light of the results reported over the last few years, this image is gradually evolving toward a much more dynamic model:

(A) *Heterogeneity in HSC proliferation and self-renewal* Analyses of the mouse and human HSC compartments have revealed variability in the cell cycle status and self-renewal capacity of individual HSCs. In the mouse, populations of “dormant” and “activated” HSCs have been identified, which are to some extent interconvertible. Dormant HSCs divide at very slow rates in homeostasis (every 145 days), exhibit the highest self-renewal and multilineage repopulation activity and, although transiently activated by bone marrow injury or by granulocyte colony-stimulating factor (G-CSF), they revert to quiescence after reestablishment of homeostasis in a non-stochastic fashion [2]. HSCs switch from a proliferative to a quiescent status 3–4 weeks after birth [3] and are believed to undergo a similar transition after ex vivo manipulation requiring cytokine stimulation (e.g., retroviral transfer). Human HSCs also show heterogeneous self-renewal ability in xenotransplantation assays, with a limited number of clones providing long-term reconstitution and others exhibiting fluctuating contributions to hematopoiesis [4].

These observations may suggest that HSC fate is initially unpredictable and occurs stochastically, but it may also in part reflect limitations in the methods currently used for the isolation and functional analysis of HSCs. Thus, despite significant improvements, the best combinations of phenotypic markers in the mouse reach about 50 % purity, measured by competitive transplant assays [1, 5], or exclude a substantial fraction of HSCs [6]. Recently, based on combined transcriptomic and functional analysis at the single cell level, Wilson et al. [7] have proposed an improved sorting strategy that increases purity up to 67 %. Strategies to isolate human HSCs still lag behind in terms of purity, and their functional validation is complicated by the relatively low engraftment frequency of xenotransplant assays. Only 9.5 % of lin- CD34+ CD38− CD45A− Thy1+ CD49f+ cells exhibit long-term repopulating activity in intrafemorally injected NOD-*scid*-*IL2Rgc* ^−/−^ mice, and a small fraction of HSCs is apparently Thy1- [8]. Therefore, even after selecting cell populations with the best combinations of markers available, studies at non-clonal levels must take into account that these cell populations are not pure but only enriched in the expected HSCs.

(B) *Heterogeneity in multilineage differentiation capacity of HSCs* Retrospective analyses of single cells or clonal transplant experiments have demonstrated different kinetics and patterns of multilineage haematopoietic reconstitution derived from individual murine HSCs. Up to 16 distinct differentiation patterns were identified, based on their relative lymphomyeloid output and kinetics [9–11]. In the absence of markers that would allow their prospective isolation, it remains unknown whether each HSC was deterministically imprinted with a differentiation program, whether cell fate choice occurred stochastically or whether it was imposed by the microenvironment upon transplantation. Moreover, there was variability in the stability or “memory” of such program: while sometimes the differentiation pattern was preserved upon serial transplantations, in other cases a switch was observed. In this regard, there is some evidence that spontaneous, stochastic gene expression “noise” in HSCs may affect lineage choice [12].

(C) *Contribution of hematopoietic progenitor cells to hematopoiesis* Traditionally, transplantation assays have served as the gold standard to assess HSC function. Under transplantation conditions, long-term reconstitution ability seems to be restricted to a small number of primitive LT-HSCs, and hematopoiesis is typically oligoclonal. However, recent studies using genetic labeling and clonal tracing of HSC have revealed a very different situation during adult steady-state hematopoiesis, in which blood cell production is highly polyclonal and predominantly maintained by “short-term HSCs” or progenitors downstream of LT-HSCs, with strong myeloid bias [13, 14]. Moreover, within myeloid progenitors, multiple subgroups with heterogeneous differentiation patterns have been identified [15].

Notwithstanding technical limitations, these data suggest that, contrasting the concept of stable and discrete HSPC populations, a more dynamic situation may exist in which there is some degree of plasticity in the proliferation and differentiation capacity of HSCs and their progeny. It is still unclear to what extent this is regulated through dynamic interactions with the microenvironment or via stochastic, cell-autonomous fluctuations in the HSC transcriptome.

## The HSC niches: structurally organized or stochastic/variable entity?

**Fig. 1:**
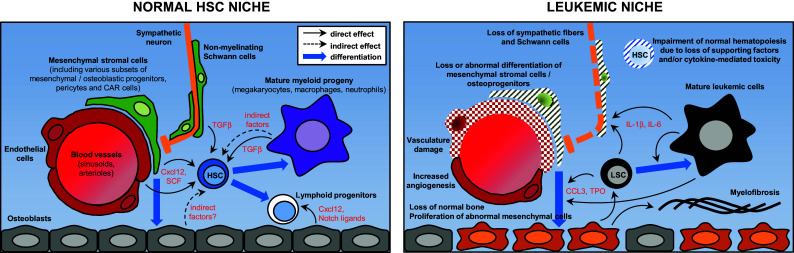
Overview of the main components of the HSC niche and their alterations in leukemia. Simplified schematic of the normal HSC niche (*left*) and its alterations in the context of malignancy. The diagram does not attempt to comprehensively include every cell population and molecule implicated in HSC regulation but to illustrate some of the best characterized candidate niche cells and factors, particularly those that have been found altered in leukemias. The *right panel* summarizes niche abnormalities observed in various experimental models representing different leukemia types. Therefore, it does not intend to propose a general model nor to describe the pathophysiology of any particular malignancy. *HSC* hematopoietic stem cell, *LSC* leukemia stem/initiating cell

The growing amount of information concerning the HSC niche in recent years has paradoxically led to a situation of relative confusion, in which virtually every non-hematopoietic cell type in the bone marrow (osteoblasts, osteocytes, endothelial cells, stromal reticular cells, pericytes including MSC-like cells, adipocytes, non-myelinated Schwann cells, sympathetic neurons) plus several mature hematopoietic populations (macrophages, neutrophils, osteoclasts, megakaryocytes, T_reg_ cells) have been proposed as niche components and/or critical regulators of HSC function (Fig. 1 left; Table 1). Here it would be worth attempting a clarification between those effects likely due to direct interaction between HSCs and anatomically close cellular components (thus properly called “niche” cells) and non-specific effects arising from the disruption of a very interconnected, complex multicellular system like the bone marrow, which requires a finely regulated homeostatic balance. Altering any major population in the bone, or the bone metabolism itself, will likely have indirect consequences on most bone marrow-resident cells due to profound structural and biochemical changes; on the other hand, experimental disruption of mature hematopoietic cells is bound to elicit a compensatory response in primitive HSPCs. However, neither of these are necessarily indicative of a “niche” function, and experimental dissection of direct and indirect effects may not be trivial.

**Table 1:** Summary of different cell types and their role in the normal and leukemic HSC niches

	Cell type	Factors produced	Role in the normal HSC niche	Role in the leukemic niche
Nonhematopoietic cells	Osteoprogenitors/osteoblasts^a^	Cxcl12, Angpt1, DLL4	Initial—but controversial—evidence implicating osteoblasts in HSC regulation (probably indirect) [18]; HSC home near, but rarely adjacent to, osteoblasts; possible role in the formation of the HSC niche; support of early lymphoid progenitors in the BM [29–31]	Diverse genetic manipulations of osteolineage cells (*Dicer1, Ctsb, Sbds, Ctnnb1*) induce preleukemic conditions [55, 60, 61]; transplanted human ALL/AML cells home near the endosteum [66–69]; CML cells stimulate production of abnormal osteoblasts that support LSC [73]
	Osteocytes	G-CSF (indirect)	Regulation of osteoblast function via cellular processes and gap junctions [20–23]	
	Sympathetic neurons	Noradrenaline	Regulate circadian egress of HSC and expression of HSC maintenance genes in MSC [47]	Production of IL-1β by JAK2^V617F^+ MPN cells leads to neuroglial damage in the BM, with loss of sympathetic fibers and associated Schwann cells; this, in turn, compromises survival of Nes+ cells [76]
	Nonmyelinating Schwann cells^a^	Active TGF-β	Maintenance of HSC quiescence [24]	
	Endothelial cells^a^	Cxcl12, Scf, Angpt1, Notch ligands	HSC maintenance/retention; deletion of *Cxcl12* or *Scf* from Tie2+ endothelial cells depletes HSC [1, 5, 16, 17]	Increased numbers of endothelial cells and angiogenesis in mice and patients with AML
	Arteriolar endothelium and pericytes^a^		Proposed role in the maintenance of HSC quiescence [25], recently questioned	Proposed problem of MSC differentiation contributing to reduced number of osteoblasts in AML
	CAR cells^a^	Cxcl12	Heterogeneous stromal cells with high Cxcl12 expression; *Cxcl12* deletion leads to HSC depletion [81]	
	Nes+ cells^a^	Cxcl12, Scf, Angpt1…	Promote homing and maintenance of HSC; mediate sympathetic circadian signaling to HSC [82]	Apoptosis of Nes+ cells in JAK2^V617F^+ MPN accelerates disease progression [76]
	Lepr+ perivascular stromal cells	Cxcl12, Scf	HSC maintenance/retention; deletion of *Cxcl12* or *Scf* from Lepr+ perivascular cells depletes HSCs [16, 17]	
	Adipocytes	Adiponectin, TNF-α	Negative regulators of homeostatic and post-transplant hematopoiesis [83]	
Hematopoietic cells	Monocytes/macrophages^a^	Vcam1, PGE2; regulate Cxcl12 on stromal cells	Promote retention of HSC and progenitors in the BM; required for HSC maintenance and erythropoiesis in response to stress [32–36]	Altered secretory pattern of leukemic myeloid cells in CML/MPN (including proinflammatory cytokines such as IL-1β and IL-6) creates abnormal niches that favors malignant over normal hematopoiesis; IL-6 also drives myeloid differentiation of MPP [73–76]
	Neutrophils	(Indirect, mediated by macrophages)	Clearance of aged neutrophils attracts macrophages; decreases CAR cells and Cxcl12 levels [47]	
	Osteoclasts		Controversial; possible role in HSPC mobilization [37–40]	
	Megakaryocytes^a^	TGF-β1, Cxcl4, PDGF-BB	Maintain HSC quiescence [41–44]; promote niche remodeling and osteoblast expansion after irradiation [45]	
	Treg cells^a^	IL-10	Locate near HSC at endosteum and protect them from immune clearance, suppressing T-cell activation [48]	

^a^Physical proximity to an HSC-enriched population in the bone marrow was directly demonstrated. Mesenchymal cell populations currently classified under different nomenclature (osteoprogenitors, Nes+ cells, Lepr+ cells, CAR cells) may partially or substantially overlap; conversely, a given cell type (e.g., endothelial) may comprise different populations depending on the choice of genetic drivers or surface markers in each particular study

One of the most controversial aspects in the last decade has been the role of osteolineage cells as HSC niche components. Although this was the first population in the BM linked to HSC regulation, and a fraction of HSCs tend to home to the vicinity of osteoblasts, some of the effects observed may be indirect, and deletion of key HSC-supporting factors from osteoblasts seems to have little effect on HSCs ([16, 17]; reviewed in [18]). One caveat of some of these studies is the difficulty to discriminate in vivo between osteoblasts/osteoprogenitors and more primitive mesenchymal progenitors, because genetic drivers routinely used to mark these populations show overlapping expression patterns (reviewed in [19]). Recent research suggests that the periosteal region may support more committed progenitors, particularly of the lymphoid lineages (see below).

Osteocytes have been reported to regulate hematopoiesis through both secretion [20] and responsiveness [21] to G-CSF. However, due to their anatomical location, effects on HSCs could be indirect. An emerging concept is that osteocytes communicate with endosteal osteoblasts through dendritic processes and may regulate their function via gap junctions [22, 23]. Mice deficient for Gsα in osteocytes exhibit loss of trabecular and cortical bone associated with expansion of myeloid cells, an effect partly mediated by G-CSF production by osteocytes [20]. In turn, osteocytes were shown to rapidly respond to G-CSF by downregulating bone-specific genes and undergoing morphological changes, with loss of the cellular processes that connect them with osteoblasts [21]. In this study, ablation or disruption of the osteocyte network preceded loss of osteoblasts, prevented G-CSF-induced HSPC mobilization, increased bone marrow Cxcl12 levels and induced HSC quiescence.

Non-myelinating Schwann cells have been reported to contribute to maintain HSCs in a quiescent state through TGF-β production [24]. Arterioles preferentially located near the bone surface were also found to provide niches for quiescent HSCs [25]. However, a recent study has found most quiescent and non-quiescent HSCs far from bone and arterioles and close to sinusoidal vessels, which are much more abundant and widely distributed in the BM [1]. Whereas most HSCs might reside in this larger region of the BM, it remains possible that a different microenvironment in the endosteum maintains a different HSC state/population. It must be kept in mind that studies reporting HSC localization in the BM have employed different marker combinations to identify non-identical HSC-enriched cellular pools, which may account for some of the discrepancies. Thus, the latter report utilized the newly described reporter α-catulin [1], while the arteriole study used SLAM markers [25].

One of the puzzling aspects when trying to extrapolate the concept of stem cell niche (as derived from evidence in solid tissues) to the bone marrow is the apparent absence of compartmentalization of the latter into discrete structures sheltering the HSC. The existence of such compartments, however, has been speculated. Structures termed “hemospheres” (containing mesenchymal, endothelial and hematopoietic cells) were shown to be enriched in CD150+ CD58− HSCs and seemed to represent sites of clonal hematopoietic cell proliferation. Hemospheres were found in sinusoidal vessels at the periphery of the BM cavity, associated with NG2+ Pdgfrb+ Nestin+ CD146+ pericytes, and required expression of VEGFR2 in the endothelium [26]. A recent study using high-resolution live imaging has suggested that this type of structures may not be permanent but dynamic. In zebrafish fetal hematopoietic tissue, the perivascular niche remodels upon arrival of a HSPC, forming a pocket-like compartment in which a group of endothelial cells surround an HSPC attached to a mesenchymal stromal cell. This structure seems to determine the polarity of HSC division [27]. Additional intravital imaging studies have revealed that HSCs also interact dynamically within the niche and, while their motility is restricted to small oscillations under resting conditions, they become more migratory upon activation (e.g., through acute infection), resulting in their contact with larger areas of the BM [28].

Recent evidence suggests that diverse bone marrow locations may function as niches for different hematopoietic progenitors. The evidence is stronger for lymphopoietic niches, which have been associated with IL-7-enriched bone regions [29, 30]. Also, specific deletion of Cxcl12 from osteoblasts reduces early lymphoid progenitors—a fraction of which was shown to localize at the endosteum—without affecting HSCs or myeloerythroid progenitors, suggesting a function of the so-called osteoblastic niche in regulating early lymphopoiesis [16]. Depletion of osteocalcin-expressing bone cells (mature osteoblasts and osteocytes), or deletion of the Notch ligand DLL4 from the same cells, impairs the production of T-lineage-competent lymphoid progenitors in the bone marrow, resulting in decreased T-cell generation in the thymus [31].

## Regulation of the HSC niche by the mature hematopoietic progeny

Recent work has highlighted the influence of different mature hematopoietic cell subsets on the regulation of the HSC niche. Among mature bone marrow cells, the greatest body of evidence implicates monocytes/macrophages as potential HSC regulators, usually indirectly through interactions with other components of the niche [32–36]. Different studies have highlighted a role of monocytes/macrophages in HSPC mobilization through regulation of osteoblastic cells. G-CSF was shown to deplete bone marrow monocytic cells, including endosteal macrophages (osteomacs). In vivo depletion of macrophages caused loss of osteoblasts and mobilization of HSPCs. Notably, expression of G-CSF receptor in monocytic cells was sufficient to induce HSPC mobilization, osteoblast suppression and Cxcl12 downregulation, suggesting that G-CSF signaling in macrophages represses some osteoblast-supporting factors [33, 36]. A population of CD169+ macrophages was shown to promote HSPC retention in the bone marrow. Experimental depletion of macrophages enhances physiological or enforced HSPC mobilization, associated with a decrease in Cxcl12 levels and downregulation of HSC maintenance genes in nestin+ cells (but not in osteoblasts) [34]. In addition, CD169+ macrophages were implicated in regulating erythropoiesis by mediating erythroblast retention in the bone marrow. Although dispensable under homeostasis, macrophages were required for a proficient erythropoietic stress response, an effect mediated by VCAM1 expression by macrophages [35]. A different study identified a population of α-SMA+ activated monocytes and macrophages that localized adjacent to HSPCs and contributed to protect HSPC from exhaustion under stress [32]. Again, the effect was partially dependent on regulation of Cxcl12 expression by stromal cells, although macrophages were also proposed to directly protect HSCs from oxidative damage by PGE2 production.

On the other hand, the influence of osteoclasts**—**which also differentiate from the monocyte/macrophage lineage—on HSCs through bone remodeling has remained controversial; different studies have reported that osteoclasts are either necessary, dispensable or even inhibitory for HSC maintenance and mobilization. Thus, some studies have suggested that osteoclasts promote the formation of BM niches [37]. Also, osteoclast inhibition with calcitonin decreased homeostatic and G-CSF-enforced HPSC mobilization [38], but their suppression by zoledronate actually enhanced G-CSF mobilization of HSPCs [36]. However, different strains of osteoclast-deficient (osteopetrotic) mice had normal or increased HSPC mobilization [39]. Also, the BM of osteoclast-deficient, osteopetrotic mice (oc/oc or RANK knockout mice) contained normal HSC activity [40].

It has been recently shown that HSCs frequently localize in the vicinity of megakaryocytes, and ablation of megakaryocytes leads to cell cycle entry of quiescent HSC, suggesting that this cellular population contributes to maintaining HSC quiescence. This interaction was reported as being mediated by soluble factors such as TGF-β1, Cxcl4 and CLEC-2-mediated thrombopoietin production [41–44]. Megakaryocytes were also shown to participate in HSC niche remodeling after radioablation. Irradiation induces megakaryocyte migration to the endosteum mediated by thrombopoietin and CD41, and PDGF-BB secreted by megakaryocytes mediates osteoblast expansion. Stimulation of megakaryocyte function by thrombopoietin administration improves HSC engraftment after transplantation [45].

Neutrophil clearance has been proposed as an additional regulatory mechanism of the HSC niche [46]. Infiltration and elimination of aged CD62^LO^ CXCR4^HI^ neutrophils in the bone marrow was proposed to cause a reduction in the size and function of the HSC niche by decreasing the number of CXCL12-abundant reticular (CAR) cells and the levels of Cxcl12, thus promoting the circadian egress of HSPCs into circulation. However, circadian oscillations of Cxcl12 expression, directly induced in bone marrow stromal cells by oscillating sympathetic activity [47], might also contribute to some of the observed changes. The reported effects of neutrophils on the HSC niche were ultimately mediated by bone marrow macrophages—which attract and phagocytose aged neutrophils—and required activation of LXR in these cells.

T_reg_ lymphocytes colocalize with HSCs near the endosteal surface in the bone marrow and contribute to protecting transplanted allogeneic HSCs from immune clearance, suggesting that the niche does not only nurture HSCs but also provides them with immune privilege [48].

## The malignant niche: a few knowns and many unknowns

Although it has long been recognized that leukemia/lymphoma development is associated with an abnormal tissue microenvironment in the affected organs, only in recent years has experimental work begun to dissect specific roles of the HSC niche in leukemogenesis (Fig. 1 right; Table 1). Progress in this direction so far has lagged behind the gradual understanding of the physiological HSC niche and shared similar technical limitations. On one hand there are uncertainties derived from the incomplete specificity, efficiency and characterization of genetic drivers used to target stromal cell populations (reviewed in [19]). These problems add to the intrinsic limitations of murine cancer models, e.g., how faithfully they recapitulate the human diseases (regarding both their hematopoietic and microenvironmental abnormalities) or how much the experimental procedure itself perturbs the niche (particularly in assays where myeloconditioning is necessary, such as xenotransplantation).

Two major ideas have emerged, supported by growing evidence: (1) genetic alterations of the niche, rather than the hematopoietic cell, may represent driving mutations during malignant transformation; (2) cancer cells remodel their niche into an abnormal environment that provides preferential support of malignant cells—in detriment of normal hematopoiesis—protects cancer cells from therapy-induced cell death, and/or drives disease progression. From these concepts immediately follows the idea that manipulating the leukemic niche might represent an advantageous therapeutic strategy, particularly in malignancies for which targeting the hematopoietic cells has proven inefficient.

### Niche abnormalities as potential initiating events

Initial studies showed that certain genetic alterations that affected broadly the BM microenvironment also promoted the development of abnormal hematopoiesis. Mice usually developed a myeloproliferative-like disease characterized by hypergranulopoiesis, extramedullary hematopoiesis, increased myeloid progenitors and eventually bone marrow failure and anemia. These phenotypes were observed upon deletion of retinoic acid receptor gamma (microenvironment dependent) [49], deletion of retinoblastoma protein (simultaneously required in microenvironment and myeloid cells) [50], ubiquitous deletion of IkBα (non-hematopoietic cell autonomous, but it is unclear whether the alteration in the microenvironment was sufficient) [51], deletion of the Notch ligand endocytosis regulator Mib1 (causing defective Notch activation in the microenvironment) [52] and haploinsufficiency of Crebbp in the stromal cells [53]. These phenotypes were associated with histological changes in the BM, such as loss of trabecular bone [49, 50, 53] or decreased number of adipocytes [52]. Among the molecular pathways implicated in these processes were increased TNFα/NFkB and aberrant Notch signaling. In these studies—more extensively discussed in recent reviews [54]—widespread deletion of the gene of interest did not allow dissection of the cell population(s) responsible for the phenotype.

Subsequent studies refined this concept by modifying specific stromal cell subsets. Specific deletion of the RNA processing enzyme *Dicer1* under the regulatory elements of *Osterix*, a gene required for osteoblastic differentiation—but not in mature osteoblasts, targeted by the regulatory elements of Osteocalcin promoter—did not only impair osteoblast differentiation, but was also found to cause a myelodysplastic syndrome characterized by dysplastic morphology, lymphoid-deficient hematopoiesis and increased HSPC proliferation and apoptosis. Key aspects of the phenotype were recapitulated by specific Osterix-cre-mediated deletion of *Sbds*, a Dicer-regulated gene that is mutated in some leukemias [55]. However, it should be noted that, although *Osterix* was originally reported to label only cells committed to become osteoblasts [56], a much wider recombination has been observed more recently after the development of stronger reporter lines [57–59] (see [19] for a review). Therefore, some of these phenotypes might be derived from gene excision in other cells, besides osteoprogenitors.

VEGF overexpression in *Col2.3*-*cre*+ osteochondroprogenitors and their progeny constitutively activated β-catenin and caused hematological abnormalities, extramedullary hematopoiesis and HSPC mobilization, likely related to the dramatic bone mass increase and the aberrant vascularization [60]. More dramatically, direct expression of a constitutively active β-catenin form in osteoblasts (*Ctnnb1* ^*CAosb*^) impaired the differentiation of hematopoietic progenitors and caused accumulation of immature myeloid cells and dysplastic neutrophils in hematopoietic tissues, resembling acute myeloid leukemia (AML). These changes were caused by expression of jagged1 in osteoblasts and consequent excessive Notch signaling in HSPCs [61].

These studies demonstrated that genetic alterations in the microenvironment can trigger a pre-leukemic condition (analogous to an oncogenic “first hit”), but at the same time suggested that additional mutations are required in the hematopoietic cells to induce malignancy and/or overt leukemia. Evidence of malignant transformation was limited in most studies. In the case of *Dicer1*-deleted mice, rare sporadic occurrences of AML that exhibited cytogenetic alterations were reported [55]. Most remarkably, frequent recurrent chromosomal alterations and somatic mutations were detected in *Ctnnb1* ^*CAosb*^ mice, including those in an ortholog region of human chromosome 7q, which is altered in AML and myelodysplastic syndromes. Notably, transformed HSCs from *Ctnnb1* ^*CAosb*^ mice became autonomous and could transfer the AML to WT recipients [61].

In spite of these experimental models providing a proof of concept, we are still lacking direct demonstration that an initial lesion in the microenvironment may play a causative role in human leukemias. However, the situation is somewhat reminiscent of that traditionally observed in extraganglionar lymphomas associated with local infectious processes (such as *Helicobacter pylori* in MALT lymphomas) in which the pathogen may provide antigenic stimulation to malignant B-cells and the tumor remits after eradication of the infection [62]. In leukemias, available evidence is only indirect. Decreased expression of *DICER*, *DROSHA* and *SBDS* was detected in MSCs—but not in leukocytes—from MDS patients compared to healthy individuals [63]. *SBDS* is inactivated by mutations in the human Shwachman–Bodian–Diamond syndrome, featuring skeletal abnormalities, bone marrow failure and susceptibility to developing myelodysplastic syndrome and secondary AML [55]. *CREBPP* heterozygosity in humans causes the Rubinstein–Taybi syndrome, characterized by skeletal defects and cancer predisposition, including leukemias [64]. Activation of β-catenin in osteoblasts, accompanied by Notch activation in hematopoietic cells, was detected in over one-third of patients with myelodysplastic syndrome or AML [61].

### Malignant cells create aberrant niches

Knowledge of how leukemic cells interact with their microenvironment has followed progress in the characterization of the normal HSC niche, and is affected by similar—if more notorious—uncertainties.

Xenografted primary human AML stem cells (CD34^+^ CD38^−^) were reported to exhibit preferential homing and engraftment to the endosteal, osteoblast-rich area of the BM [65], while acute lymphoblastic leukemia (ALL) cells tended to localize to vascular regions expressing E-selectin and Cxcl12, overlapping with perivascular HSPC niches [66, 67]; however, it was later recognized that these endothelial microdomains were juxtaposed to the endosteum [68], suggesting similar primary homing location of AML/ALL cells. Endosteal human leukemia stem cells (LSCs) causing AML were reported as characteristically quiescent and chemoresistant; cell cycle entry induced by G-CSF enhanced chemotherapy-induced apoptosis and elimination of LSCs [65, 69].

Xenotransplantation of ALL cell lines has been shown to disrupt the normal bone marrow microenvironment [66, 68]. Particularly, these cells can cause severe damage of the vasculature and endosteum-lining cells and lead to the formation of abnormal niches primarily formed by the mutated cells, which highly express stem cell factor but produce low levels of Cxcl12. In these aberrant niches, both the numbers and the traffic of normal HSPCs were reduced. This was proposed as a mechanism by which normal hematopoiesis could be impaired even in the presence of a low tumor burden. In the same xenotransplant model, chemotherapy was shown to induce the formation of transient niches consisting of small foci of surviving ALL cells and nestin+ LepR+ NG2+ stromal cells with properties of MSCs (multipotent sphere formation, in vitro differentiation). Formation of these structures required CCL3 and TGFb1 produced by ALL cells and, in spite of their short life, were proposed to protect ALL cells from chemotherapy-induced apoptosis. A recent study has shown that T-ALL cells require Cxcl12-producing endothelial cells, and CXCR4 antagonism suppressed T-ALL in primary xenografts [70].

It has been recently shown that MSCs exhibit an altered gene expression program in myelodysplastic syndromes, with increased expression of genes associated with osteoprogenitor cell fate, inflammation and fibrosis. These MSCs are capable of promoting the engraftment or propagation of myelodysplasia in orthotopic xenografts [71].

Severe osteoblastic defects were found in a model of myeloid blast-crisis chronic myeloid leukemia (CML, driven by *BCR*-*ABL;Nup98/HoxA9*), with decreased osteoprogenitors, endosteal-lining osteoblasts and bone mass, associated with increased CCL3 expression [72]. Using a mouse model of chronic-phase, BCR-ABL-induced CML (*SCL*-*tTA;TRE*-*BCR/ABL* mice), two studies have provided evidence for the concept that leukemic cells can remodel the bone marrow environment into a self-supporting niche that impairs normal hematopoiesis. Leukemic cells were shown to stimulate excessive production of osteoblastic cells. These cells had reduced expression of HSC maintenance factors and impaired ability to support normal HSCs, while having limited impact on LSCs. Abnormal osteoblastic cells overexpressed inflammation- and myelofibrosis-related genes, contributed to bone marrow fibrosis, and their expansion was driven by TPO, CCL3 and direct contact with leukemic cells [73]. In another study, G-CSF overproduction by CML cells reduced Cxcl12 expression by bone marrow stromal cells and increased several pro-inflammatory cytokines, leading to selective impairment of normal HSCs that also favored the growth of CML stem cells [74]. Pro-inflammatory cytokines produced by mature leukemic cells had also direct effects on the leukemic HSPC; thus, BCR-ABL-dependent secretion of IL-6 by CML cells drives myeloid differentiation of leukemic multipotent MPP, establishing a feed-forward loop [75].

Recent work from our laboratory has demonstrated that the manifestation of *JAK2* ^*V617F*^-induced myeloproliferative neoplasms (MPN) requires the loss of sympathetic regulation in the HSC niche [76]. Sympathetic fibers and associated Schwann cells are consistently decreased in the bone marrow of *JAK2* ^*V617F*^-positive MPN patients and mice due to apoptosis induced by MPN cell-derived IL-1β. In turn, neuroglial damage compromises the survival of nestin^+^ cells, which are also reduced in number over the course of the disease. This loss of MSCs plays a driving role in MPN development, since selective depletion of nestin^+^ cells, or Cxcl12 produced by them, is sufficient to accelerate disease progression. Conversely, pharmacological compensation of neural damage blocks MPN progression (see below).

### The leukemic niche as a therapeutic target

Growing evidence of the active participation of the HSC niche in hematological malignancies, either by providing an initiating lesion or by responding to leukemia-induced remodeling, suggests that its therapeutic targeting may have therapeutic benefit. This could be potentially achieved through inhibition of essential interactions between LSCs and supporting cells, or by reversion of niche-related changes that drive the course of the disease. We have provided direct evidence of the latter in the context of *JAK2* ^*V617F*^-induced MPN, in which loss of nestin+ MSCs caused by neuroglial damage promotes disease progression [76]. Rescue of the defective sympathetic stimulation by chronic administration of β3-adrenergic agonists prevented the loss of nestin^+^ MSC and inhibited disease progression at both early and late stages, preventing neutrophilia, thrombocytosis, IL-1β production, bone marrow fibrosis and expansion of LSCs, while having negligible effects on normal HSCs. Similarly, treatment of MPN-affected mice with the neuroprotective molecule 4-methylcatecol, which protects sympathetic fibers and Schwann cells, abolished MPN-related neutrophilia. These results demonstrate that specific reversion of malignancy-induced changes in the bone marrow microenvironment may be sufficient to block MPN. They also prove that niche transformation represents a major driving force and a requirement for disease progression, and provide a novel, potential safe therapeutic approach where hematopoietic cell-directed therapies have previously shown limited efficacy.

However, this is very unlikely the case of more aggressive leukemias, such as AML, despite some similarities shown in a recent study suggesting that sympathetic neuropathy caused by acute myeloid leukemic cells promotes malignancy in an altered hematopoietic stem cell niche [77]. Unlike in MPN [76], adrenergic drugs did not significantly impact AML progression in this study. However, emerging evidence suggests that the microenvironment might represent a feasible therapeutic target for AML. For instance, inhibition of Cxcr4/Cxcl12 signaling may show some therapeutic advantage in AML. Cxcr4 antagonists (AML3100/Plerixafor and the novel LY2510924) induce leukemic cell mobilization and enhance the antileukemic action of chemotherapy in xenotransplant models [78, 79]. Furthermore, addition of AMD3100 to cytotoxic chemotherapy increased remission rates in patients with relapsed or refractory AML in a phase 1/2 clinical study [80].

## Considerations for the future

As outlined at the beginning of this review, elucidation of the cellular and molecular interactions in the HSC niche has two major practical motivations. One is the identification of key molecules that would allow the production, maintenance and expansion of HSCs. Identification of the specific factors that contribute to maintaining stemness in vivo would facilitate the development of chemically defined culture media, avoiding the use of animal-derived culture supplements and possibly the need for time-consuming coculture protocols. The identification of specific microenvironments and factors required for the maintenance of different types of hematopoietic progenitors could make possible the selective ex vivo generation of different lineages for specific applications.

Another aspect with translational potential is the identification of alterations in the bone marrow niche directly associated with, and required for, the development of each type of leukemia. It is expected that different malignancies will induce a specific set of abnormalities and will also differ in their dependence on the niche. Thus, it would not be surprising to find that aggressive, acute leukemias with accumulated mutations may become relatively cell autonomous and less sensitive to microenvironmental regulation. Most relevant will be to identify cellular processes or pathways selectively required for the maintenance of malignant progenitors—as opposed to normal HSCs—and susceptible to pharmacological targeting. The success of niche-directed therapies will likely depend on how accurately we can validate preclinical findings from mouse models in the human system, for which it will be critical to develop parallel sets of molecular markers for human HSCs and niche cells, as well as more faithful xenotransplant models.

From the more theoretical point of view, research in the immediate future is expected to refine our knowledge of the composition of the HSC niche, dissecting between direct interactions with *bona fide* “niche cells” and indirect signals from more distant components. It would also be advantageous to clarify some of the confusion in the field resulting from the use of incompletely characterized genetic drivers and partially overlapping markers (reviewed in [19]). Again, it is also expected that improved xenograft models and human cell markers will allow a more direct investigation of the biology of the human HSC niche.

## Abbreviations

HSC: Hematopoietic stem cell
HSPC: Hematopoietic stem cells and progenitors
MPP: Multipotent progenitors
LSC: Leukemia stem cell
MSC: Mesenchymal stem cell
AML: Acute myeloid leukemia
ALL: Acute lymphoblastic leukemia
MPN: Myeloproliferative neoplasm
CML: Chronic myeloid leukemia

## References

[1] Acar M, Kocherlakota KS, Murphy MM, Peyer JG, Oguro H, Inra CN, Jaiyeola C, Zhao Z, Luby-Phelps K, Morrison SJ (2015). Deep imaging of bone marrow shows non-dividing stem cells are mainly perisinusoidal. Nature.

[2] Wilson A, Laurenti E, Oser G, van der Wath RC, Blanco-Bose W, Jaworski M, Offner S, Dunant CF, Eshkind L, Bockamp E, Lio P, Macdonald HR, Trumpp A (2008). Hematopoietic stem cells reversibly switch from dormancy to self-renewal during homeostasis and repair. Cell.

[3] Bowie MB, McKnight KD, Kent DG, McCaffrey L, Hoodless PA, Eaves CJ (2006). Hematopoietic stem cells proliferate until after birth and show a reversible phase-specific engraftment defect. J Clin Invest.

[4] McKenzie JL, Gan OI, Doedens M, Wang JC, Dick JE (2006). Individual stem cells with highly variable proliferation and self-renewal properties comprise the human hematopoietic stem cell compartment. Nat Immunol.

[5] Kiel MJ, Yilmaz OH, Iwashita T, Terhorst C, Morrison SJ (2005). SLAM family receptors distinguish hematopoietic stem and progenitor cells and reveal endothelial niches for stem cells. Cell.

[6] Matsuzaki Y, Kinjo K, Mulligan RC, Okano H (2004). Unexpectedly efficient homing capacity of purified murine hematopoietic stem cells. Immunity.

[7] Wilson NK, Kent DG, Buettner F, Shehata M, Macaulay IC, Calero-Nieto FJ, Sanchez Castillo M, Oedekoven CA, Diamanti E, Schulte R, Ponting CP, Voet T, Caldas C, Stingl J, Green AR, Theis FJ, Gottgens B (2015). Combined single-cell functional and gene expression analysis resolves heterogeneity within stem cell populations. Cell Stem Cell.

[8] Notta F, Doulatov S, Laurenti E, Poeppl A, Jurisica I, Dick JE (2011). Isolation of single human hematopoietic stem cells capable of long-term multilineage engraftment. Science.

[9] Sieburg HB, Cho RH, Dykstra B, Uchida N, Eaves CJ, Muller-Sieburg CE (2006). The hematopoietic stem compartment consists of a limited number of discrete stem cell subsets. Blood.

[10] Dykstra B, Kent D, Bowie M, McCaffrey L, Hamilton M, Lyons K, Lee SJ, Brinkman R, Eaves C (2007). Long-term propagation of distinct hematopoietic differentiation programs in vivo. Cell Stem Cell.

[11] Lu R, Neff NF, Quake SR, Weissman IL (2011). Tracking single hematopoietic stem cells in vivo using high-throughput sequencing in conjunction with viral genetic barcoding. Nat Biotechnol.

[12] Chang HH, Hemberg M, Barahona M, Ingber DE, Huang S (2008). Transcriptome-wide noise controls lineage choice in mammalian progenitor cells. Nature.

[13] Busch K, Klapproth K, Barile M, Flossdorf M, Holland-Letz T, Schlenner SM, Reth M, Hofer T, Rodewald HR (2015). Fundamental properties of unperturbed haematopoiesis from stem cells in vivo. Nature.

[14] Sun J, Ramos A, Chapman B, Johnnidis JB, Le L, Ho YJ, Klein A, Hofmann O, Camargo FD (2014). Clonal dynamics of native haematopoiesis. Nature.

[15] Paul F, Arkin Y, Giladi A, Jaitin DA, Kenigsberg E, Keren-Shaul H, Winter D, Lara-Astiaso D, Gury M, Weiner A, David E, Cohen N, Lauridsen FK, Haas S, Schlitzer A, Mildner A, Ginhoux F, Jung S, Trumpp A, Porse BT, Tanay A, Amit I (2015). Transcriptional heterogeneity and lineage commitment in myeloid progenitors. Cell.

[16] Ding L, Morrison SJ (2013). Haematopoietic stem cells and early lymphoid progenitors occupy distinct bone marrow niches. Nature.

[17] Ding L, Saunders TL, Enikolopov G, Morrison SJ (2012). Endothelial and perivascular cells maintain haematopoietic stem cells. Nature.

[18] Morrison SJ, Scadden DT (2014). The bone marrow niche for haematopoietic stem cells. Nature.

[19] Mendez-Ferrer S, Scadden DT, Sanchez-Aguilera A (2015). Bone marrow stem cells: current and emerging concepts. Ann N Y Acad Sci.

[20] Fulzele K, Krause DS, Panaroni C, Saini V, Barry KJ, Liu X, Lotinun S, Baron R, Bonewald L, Feng JQ, Chen M, Weinstein LS, Wu JY, Kronenberg HM, Scadden DT, Divieti Pajevic P (2013). Myelopoiesis is regulated by osteocytes through Gsalpha-dependent signaling. Blood.

[21] Asada N, Katayama Y, Sato M, Minagawa K, Wakahashi K, Kawano H, Kawano Y, Sada A, Ikeda K, Matsui T, Tanimoto M (2013). Matrix-embedded osteocytes regulate mobilization of hematopoietic stem/progenitor cells. Cell Stem Cell.

[22] Kamioka H, Honjo T, Takano-Yamamoto T (2001). A three-dimensional distribution of osteocyte processes revealed by the combination of confocal laser scanning microscopy and differential interference contrast microscopy. Bone.

[23] Taylor AF, Saunders MM, Shingle DL, Cimbala JM, Zhou Z, Donahue HJ (2007). Mechanically stimulated osteocytes regulate osteoblastic activity via gap junctions. Am J Physiol Cell Physiol.

[24] Yamazaki S, Ema H, Karlsson G, Yamaguchi T, Miyoshi H, Shioda S, Taketo MM, Karlsson S, Iwama A, Nakauchi H (2011). Nonmyelinating Schwann cells maintain hematopoietic stem cell hibernation in the bone marrow niche. Cell.

[25] Kunisaki Y, Bruns I, Scheiermann C, Ahmed J, Pinho S, Zhang D, Mizoguchi T, Wei Q, Lucas D, Ito K, Mar JC, Bergman A, Frenette PS (2013). Arteriolar niches maintain haematopoietic stem cell quiescence. Nature.

[26] Wang L, Benedito R, Bixel MG, Zeuschner D, Stehling M, Savendahl L, Haigh JJ, Snippert H, Clevers H, Breier G, Kiefer F, Adams RH (2013). Identification of a clonally expanding haematopoietic compartment in bone marrow. EMBO J.

[27] Tamplin OJ, Durand EM, Carr LA, Childs SJ, Hagedorn EJ, Li P, Yzaguirre AD, Speck NA, Zon LI (2015). Hematopoietic stem cell arrival triggers dynamic remodeling of the perivascular niche. Cell.

[28] Rashidi NM, Scott MK, Scherf N, Krinner A, Kalchschmidt JS, Gounaris K, Selkirk ME, Roeder I, Lo Celso C (2014). In vivo time-lapse imaging shows diverse niche engagement by quiescent and naturally activated hematopoietic stem cells. Blood.

[29] Tokoyoda K, Egawa T, Sugiyama T, Choi BI, Nagasawa T (2004). Cellular niches controlling B lymphocyte behavior within bone marrow during development. Immunity.

[30] Wu JY, Purton LE, Rodda SJ, Chen M, Weinstein LS, McMahon AP, Scadden DT, Kronenberg HM (2008). Osteoblastic regulation of B lymphopoiesis is mediated by Gs{alpha}-dependent signaling pathways. Proc Natl Acad Sci USA.

[31] Yu VW, Saez B, Cook C, Lotinun S, Pardo-Saganta A, Wang YH, Lymperi S, Ferraro F, Raaijmakers MH, Wu JY, Zhou L, Rajagopal J, Kronenberg HM, Baron R, Scadden DT (2015). Specific bone cells produce DLL4 to generate thymus-seeding progenitors from bone marrow. J Exp Med.

[32] Ludin A, Itkin T, Gur-Cohen S, Mildner A, Shezen E, Golan K, Kollet O, Kalinkovich A, Porat Z, D’Uva G, Schajnovitz A, Voronov E, Brenner DA, Apte RN, Jung S, Lapidot T (2012). Monocytes-macrophages that express alpha-smooth muscle actin preserve primitive hematopoietic cells in the bone marrow. Nat Immunol.

[33] Christopher MJ, Rao M, Liu F, Woloszynek JR, Link DC (2011). Expression of the G-CSF receptor in monocytic cells is sufficient to mediate hematopoietic progenitor mobilization by G-CSF in mice. J Exp Med.

[34] Chow A, Lucas D, Hidalgo A, Mendez-Ferrer S, Hashimoto D, Scheiermann C, Battista M, Leboeuf M, Prophete C, van Rooijen N, Tanaka M, Merad M, Frenette PS (2011). Bone marrow CD169+ macrophages promote the retention of hematopoietic stem and progenitor cells in the mesenchymal stem cell niche. J Exp Med.

[35] Chow A, Huggins M, Ahmed J, Hashimoto D, Lucas D, Kunisaki Y, Pinho S, Leboeuf M, Noizat C, van Rooijen N, Tanaka M, Zhao ZJ, Bergman A, Merad M, Frenette PS (2013). CD169(+) macrophages provide a niche promoting erythropoiesis under homeostasis and stress. Nat Med.

[36] Winkler IG, Sims NA, Pettit AR, Barbier V, Nowlan B, Helwani F, Poulton IJ, van Rooijen N, Alexander KA, Raggatt LJ, Levesque JP (2010). Bone marrow macrophages maintain hematopoietic stem cell (HSC) niches and their depletion mobilizes HSCs. Blood.

[37] Mansour A, Abou-Ezzi G, Sitnicka E, Jacobsen SE, Wakkach A, Blin-Wakkach C (2012). Osteoclasts promote the formation of hematopoietic stem cell niches in the bone marrow. J Exp Med.

[38] Kollet O, Dar A, Shivtiel S, Kalinkovich A, Lapid K, Sztainberg Y, Tesio M, Samstein RM, Goichberg P, Spiegel A, Elson A, Lapidot T (2006). Osteoclasts degrade endosteal components and promote mobilization of hematopoietic progenitor cells. Nat Med.

[39] Miyamoto K, Yoshida S, Kawasumi M, Hashimoto K, Kimura T, Sato Y, Kobayashi T, Miyauchi Y, Hoshi H, Iwasaki R, Miyamoto H, Hao W, Morioka H, Chiba K, Yasuda H, Penninger JM, Toyama Y, Suda T, Miyamoto T (2011). Osteoclasts are dispensable for hematopoietic stem cell maintenance and mobilization. J Exp Med.

[40] Flores C, Moscatelli I, Thudium CS, Gudmann NS, Thomsen JS, Bruel A, Karsdal MA, Henriksen K, Richter J (2013). Osteoclasts are not crucial for hematopoietic stem cell maintenance in adult mice. Haematologica.

[41] Bruns I, Lucas D, Pinho S, Ahmed J, Lambert MP, Kunisaki Y, Scheiermann C, Schiff L, Poncz M, Bergman A, Frenette PS (2014). Megakaryocytes regulate hematopoietic stem cell quiescence through CXCL4 secretion. Nat Med.

[42] Zhao M, Perry JM, Marshall H, Venkatraman A, Qian P, He XC, Ahamed J, Li L (2014). Megakaryocytes maintain homeostatic quiescence and promote post-injury regeneration of hematopoietic stem cells. Nat Med.

[43] Nakamura-Ishizu A, Takubo K, Kobayashi H, Suzuki-Inoue K, Suda T (2015). CLEC-2 in megakaryocytes is critical for maintenance of hematopoietic stem cells in the bone marrow. J Exp Med.

[44] Nakamura-Ishizu A, Takubo K, Fujioka M, Suda T (2014). Megakaryocytes are essential for HSC quiescence through the production of thrombopoietin. Biochem Biophys Res Commun.

[45] Olson TS, Caselli A, Otsuru S, Hofmann TJ, Williams R, Paolucci P, Dominici M, Horwitz EM (2013). Megakaryocytes promote murine osteoblastic HSC niche expansion and stem cell engraftment after radioablative conditioning. Blood.

[46] Casanova-Acebes M, Pitaval C, Weiss LA, Nombela-Arrieta C, Chevre R, Noelia AG, Kunisaki Y, Zhang D, van Rooijen N, Silberstein LE, Weber C, Nagasawa T, Frenette PS, Castrillo A, Hidalgo A (2013). Rhythmic modulation of the hematopoietic niche through neutrophil clearance. Cell.

[47] Mendez-Ferrer S, Lucas D, Battista M, Frenette PS (2008). Haematopoietic stem cell release is regulated by circadian oscillations. Nature.

[48] Fujisaki J, Wu J, Carlson AL, Silberstein L, Putheti P, Larocca R, Gao W, Saito TI, Lo Celso C, Tsuyuzaki H, Sato T, Cote D, Sykes M, Strom TB, Scadden DT, Lin CP (2011). In vivo imaging of Treg cells providing immune privilege to the haematopoietic stem-cell niche. Nature.

[49] Walkley CR, Olsen GH, Dworkin S, Fabb SA, Swann J, McArthur GA, Westmoreland SV, Chambon P, Scadden DT, Purton LE (2007). A microenvironment-induced myeloproliferative syndrome caused by retinoic acid receptor gamma deficiency. Cell.

[50] Walkley CR, Shea JM, Sims NA, Purton LE, Orkin SH (2007). Rb regulates interactions between hematopoietic stem cells and their bone marrow microenvironment. Cell.

[51] Rupec RA, Jundt F, Rebholz B, Eckelt B, Weindl G, Herzinger T, Flaig MJ, Moosmann S, Plewig G, Dorken B, Forster I, Huss R, Pfeffer K (2005). Stroma-mediated dysregulation of myelopoiesis in mice lacking I kappa B alpha. Immunity.

[52] Kim YW, Koo BK, Jeong HW, Yoon MJ, Song R, Shin J, Jeong DC, Kim SH, Kong YY (2008). Defective Notch activation in microenvironment leads to myeloproliferative disease. Blood.

[53] Zimmer SN, Zhou Q, Zhou T, Cheng Z, Abboud-Werner SL, Horn D, Lecocke M, White R, Krivtsov AV, Armstrong SA, Kung AL, Livingston DM, Rebel VI (2011). Crebbp haploinsufficiency in mice alters the bone marrow microenvironment, leading to loss of stem cells and excessive myelopoiesis. Blood.

[54] Raaijmakers MH (2012). Myelodysplastic syndromes: revisiting the role of the bone marrow microenvironment in disease pathogenesis. Int J Hematol.

[55] Raaijmakers MH, Mukherjee S, Guo S, Zhang S, Kobayashi T, Schoonmaker JA, Ebert BL, Al-Shahrour F, Hasserjian RP, Scadden EO, Aung Z, Matza M, Merkenschlager M, Lin C, Rommens JM, Scadden DT (2010). Bone progenitor dysfunction induces myelodysplasia and secondary leukaemia. Nature.

[56] Rodda SJ, McMahon AP (2006). Distinct roles for Hedgehog and canonical Wnt signaling in specification, differentiation and maintenance of osteoblast progenitors. Development.

[57] Madisen L, Zwingman TA, Sunkin SM, Oh SW, Zariwala HA, Gu H, Ng LL, Palmiter RD, Hawrylycz MJ, Jones AR, Lein ES, Zeng H (2010). A robust and high-throughput Cre reporting and characterization system for the whole mouse brain. Nat Neurosci.

[58] Liu Y, Strecker S, Wang L, Kronenberg MS, Wang W, Rowe DW, Maye P (2013). Osterix-cre labeled progenitor cells contribute to the formation and maintenance of the bone marrow stroma. PLoS One.

[59] Maes C, Kobayashi T, Selig MK, Torrekens S, Roth SI, Mackem S, Carmeliet G, Kronenberg HM (2010). Osteoblast precursors, but not mature osteoblasts, move into developing and fractured bones along with invading blood vessels. Dev Cell.

[60] Maes C, Goossens S, Bartunkova S, Drogat B, Coenegrachts L, Stockmans I, Moermans K, Nyabi O, Haigh K, Naessens M, Haenebalcke L, Tuckermann JP, Tjwa M, Carmeliet P, Mandic V, David JP, Behrens A, Nagy A, Carmeliet G, Haigh JJ (2010). Increased skeletal VEGF enhances beta-catenin activity and results in excessively ossified bones. EMBO J.

[61] Kode A, Manavalan JS, Mosialou I, Bhagat G, Rathinam CV, Luo N, Khiabanian H, Lee A, Murty VV, Friedman R, Brum A, Park D, Galili N, Mukherjee S, Teruya-Feldstein J, Raza A, Rabadan R, Berman E, Kousteni S (2014). Leukaemogenesis induced by an activating beta-catenin mutation in osteoblasts. Nature.

[62] Herreros B, Sanchez-Aguilera A, Piris MA (2008). Lymphoma microenvironment: culprit or innocent?. Leukemia.

[63] Santamaria C, Muntion S, Roson B, Blanco B, Lopez-Villar O, Carrancio S, Sanchez-Guijo FM, Diez-Campelo M, Alvarez-Fernandez S, Sarasquete ME, de las Rivas J, Gonzalez M, San Miguel JF, Del Canizo MC (2012). Impaired expression of DICER, DROSHA, SBDS and some microRNAs in mesenchymal stromal cells from myelodysplastic syndrome patients. Haematologica.

[64] Roelfsema JH, Peters DJ (2007). Rubinstein-Taybi syndrome: clinical and molecular overview. Expert Rev Mol Med.

[65] Ishikawa F, Yoshida S, Saito Y, Hijikata A, Kitamura H, Tanaka S, Nakamura R, Tanaka T, Tomiyama H, Saito N, Fukata M, Miyamoto T, Lyons B, Ohshima K, Uchida N, Taniguchi S, Ohara O, Akashi K, Harada M, Shultz LD (2007). Chemotherapy-resistant human AML stem cells home to and engraft within the bone-marrow endosteal region. Nat Biotechnol.

[66] Colmone A, Amorim M, Pontier AL, Wang S, Jablonski E, Sipkins DA (2008). Leukemic cells create bone marrow niches that disrupt the behavior of normal hematopoietic progenitor cells. Science.

[67] Sipkins DA, Wei X, Wu JW, Runnels JM, Cote D, Means TK, Luster AD, Scadden DT, Lin CP (2005). In vivo imaging of specialized bone marrow endothelial microdomains for tumour engraftment. Nature.

[68] Duan CW, Shi J, Chen J, Wang B, Yu YH, Qin X, Zhou XC, Cai YJ, Li ZQ, Zhang F, Yin MZ, Tao Y, Mi JQ, Li LH, Enver T, Chen GQ, Hong DL (2014). Leukemia propagating cells rebuild an evolving niche in response to therapy. Cancer Cell.

[69] Saito Y, Uchida N, Tanaka S, Suzuki N, Tomizawa-Murasawa M, Sone A, Najima Y, Takagi S, Aoki Y, Wake A, Taniguchi S, Shultz LD, Ishikawa F (2010). Induction of cell cycle entry eliminates human leukemia stem cells in a mouse model of AML. Nat Biotechnol.

[70] Pitt LA, Tikhonova AN, Hu H, Trimarchi T, King B, Gong Y, Sanchez-Martin M, Tsirigos A, Littman DR, Ferrando AA, Morrison SJ, Fooksman DR, Aifantis I, Schwab SR (2015). CXCL12-producing vascular endothelial niches control acute T cell leukemia maintenance. Cancer Cell.

[71] Medyouf H, Mossner M, Jann JC, Nolte F, Raffel S, Herrmann C, Lier A, Eisen C, Nowak V, Zens B, Mudder K, Klein C, Oblander J, Fey S, Vogler J, Fabarius A, Riedl E, Roehl H, Kohlmann A, Staller M, Haferlach C, Muller N, John T, Platzbecker U, Metzgeroth G, Hofmann WK, Trumpp A, Nowak D (2014). Myelodysplastic cells in patients reprogram mesenchymal stromal cells to establish a transplantable stem cell niche disease unit. Cell Stem Cell.

[72] Frisch BJ, Ashton JM, Xing L, Becker MW, Jordan CT, Calvi LM (2012). Functional inhibition of osteoblastic cells in an in vivo mouse model of myeloid leukemia. Blood.

[73] Schepers K, Pietras EM, Reynaud D, Flach J, Binnewies M, Garg T, Wagers AJ, Hsiao EC, Passegue E (2013). Myeloproliferative neoplasia remodels the endosteal bone marrow niche into a self-reinforcing leukemic niche. Cell Stem Cell.

[74] Zhang B, Ho YW, Huang Q, Maeda T, Lin A, Lee SU, Hair A, Holyoake TL, Huettner C, Bhatia R (2012). Altered microenvironmental regulation of leukemic and normal stem cells in chronic myelogenous leukemia. Cancer Cell.

[75] Reynaud D, Pietras E, Barry-Holson K, Mir A, Binnewies M, Jeanne M, Sala-Torra O, Radich JP, Passegue E (2011). IL-6 controls leukemic multipotent progenitor cell fate and contributes to chronic myelogenous leukemia development. Cancer Cell.

[76] Arranz L, Sanchez-Aguilera A, Martin-Perez D, Isern J, Langa X, Tzankov A, Lundberg P, Muntion S, Tzeng YS, Lai DM, Schwaller J, Skoda RC, Mendez-Ferrer S (2014). Neuropathy of haematopoietic stem cell niche is essential for myeloproliferative neoplasms. Nature.

[77] Hanoun M, Zhang D, Mizoguchi T, Pinho S, Pierce H, Kunisaki Y, Lacombe J, Armstrong SA, Duhrsen U, Frenette PS (2014). Acute myelogenous leukemia-induced sympathetic neuropathy promotes malignancy in an altered hematopoietic stem cell niche. Cell Stem Cell.

[78] Nervi B, Ramirez P, Rettig MP, Uy GL, Holt MS, Ritchey JK, Prior JL, Piwnica-Worms D, Bridger G, Ley TJ, DiPersio JF (2009). Chemosensitization of acute myeloid leukemia (AML) following mobilization by the CXCR4 antagonist AMD3100. Blood.

[79] Zeng Z, Shi YX, Samudio IJ, Wang RY, Ling X, Frolova O, Levis M, Rubin JB, Negrin RR, Estey EH, Konoplev S, Andreeff M, Konopleva M (2009). Targeting the leukemia microenvironment by CXCR4 inhibition overcomes resistance to kinase inhibitors and chemotherapy in AML. Blood.

[80] Uy GL, Rettig MP, Motabi IH, McFarland K, Trinkaus KM, Hladnik LM, Kulkarni S, Abboud CN, Cashen AF, Stockerl-Goldstein KE, Vij R, Westervelt P, DiPersio JF (2012). A phase 1/2 study of chemosensitization with the CXCR4 antagonist plerixafor in relapsed or refractory acute myeloid leukemia. Blood.

[81] Sugiyama T, Kohara H, Noda M, Nagasawa T (2006). Maintenance of the hematopoietic stem cell pool by CXCL12-CXCR4 chemokine signaling in bone marrow stromal cell niches. Immunity.

[82] Mendez-Ferrer S, Michurina TV, Ferraro F, Mazloom AR, Macarthur BD, Lira SA, Scadden DT, Ma’ayan A, Enikolopov GN, Frenette PS (2010). Mesenchymal and haematopoietic stem cells form a unique bone marrow niche. Nature.

[83] Naveiras O, Nardi V, Wenzel PL, Hauschka PV, Fahey F, Daley GQ (2009). Bone-marrow adipocytes as negative regulators of the haematopoietic microenvironment. Nature.

